# Xylitol toxicosis and serotonin‐like syndrome occurring simultaneously in a dog secondary to ingestion of Natrol 5‐HTP Fast Dissolve Tablets

**DOI:** 10.1002/ccr3.1869

**Published:** 2018-11-08

**Authors:** Jennifer M. Ortolani, Tara J. Bellis, Michelle D. Griego

**Affiliations:** ^1^ Critical Care Department BluePearl Veterinary Partners New York City New York

**Keywords:** canine, Natrol 5‐HTP, serotonin‐like syndrome, veterinary, xylitol toxicosis

## Abstract

Xylitol and serotonergic compounds are included in the manufacture of numerous over‐the‐counter products. Both compounds have been documented to cause toxicosis in dogs. This report details the first case of simultaneous xylitol toxicosis and serotonin‐like syndrome in a dog, secondary to ingestion of a single over‐the‐counter product.

## 1 INTRODUCTION

Five‐hydroxytryptophan (5‐HTP) is a by‐product of L‐tryptophan, an essential amino acid, and is produced commercially from Griffonia simplicifolia seeds. Tryptophan is converted to 5‐HTP via tryptophan hydroxylase, and 5‐HTP is then converted to serotonin (5‐HT; hydroxytryptamine) by L‐amino decarboxylase.^1^ Serotonin is a central nervous system neurotransmitter with additional effects on platelet aggregation and stimulatory effects on the smooth muscle of the gastrointestinal tract, respiratory tract, and cardiovascular system. Five‐hydroxytryptophan is available over‐the‐counter in a variety of formulations, and is used to enhance serotonin production, thereby assisting in the treatment of a number of conditions in people, including sleep disorders, anxiety, and obesity.^2^ The authors are aware of anecdotal reports of the use of 5‐HTP in dogs at low doses (1 mg/kg by mouth every 12 hours) to help manage fear, anxiety, and reactivity. Natrol 5‐HTP Fast Dissolve tablets (Natrol 5‐HTP Fast Dissolve tablets wild berry flavor, Natrol LLC., Chatsworth, CA) are manufactured with an artificial sweetener called xylitol. Xylitol is a 5‐carbon sugar alcohol that has gained popularity in recent years as a sugar substitute in foodstuffs for people with diabetes. It is also popular with dental health professionals for its natural anti‐cariogenic properties and has been used in wound dressings to decrease biofilm efficacy of *Pseudomonas aeruginosa* and methicillin‐resistant *Staphylococcus aureus* in dogs.^2^, ^3^ Both 5‐HTP and xylitol have been reported to cause toxicosis in dogs, however to the authors’ knowledge, this is the first report of an over‐the‐counter supplement causing simultaneous signs of both toxicities in a dog.

## 2 CASE SUMMARY

A 3‐year‐old male, castrated, Dachshund, weighing 8 kg, was presented to the primary care veterinarian following ingestion of 29 tablets of Natrol 5‐HTP Fast Dissolve Tablets. Each tablet contained 100 mg of 5‐HTP and 820 mg of xylitol, corresponding to a dose of 362.5 mg/kg 5‐HTP and 2.973 g/kg of xylitol. The ingestion was witnessed by the owner, and induction of emesis was attempted by administration of hydrogen peroxide in the home (quantity and formulation unknown). Emesis was unsuccessful, and the patient was presented to the primary care veterinarian. Upon arrival, the patient was agitated and tremoring. A full neurologic assessment was not documented, however, bilateral mydriasis was reported. The patient was ambulatory and responsive. Heart rate was 120 beats per minute (bpm), respiratory rate was 60 breaths per minute, and rectal temperature was 37.7°C (100°F) (RI 37.5‐39.2°C; 99.5−102.5°F). Mucous membranes were pink with a capillary refill time (CRT) of <2 seconds. The patient exhibited increased respiratory effort, and harsh bronchovesicular sounds were auscultated bilaterally. Oxygen saturation as measured by pulse oximetry was 95% on room air. An apomorphine tablet (2 mg tablet, 0.25 mg/kg) was administered into the right conjunctival sac. Induction of emesis was unsuccessful. Initial blood glucose (BG) was high at 9.768 mmol/L (176 mg/dL) (RI 4.4‐6.7 mmol/L; 80‐120 mg/dL). Complete blood count (CBC) values were within reference ranges. A chemistry panel demonstrated normal baseline liver enzymes. Alanine aminotransferase (ALT) was 38 IU/L (RI 12‐118 IU/L), aspartate aminotransferase (AST) was 29 IU/L (RI 15‐66 IU/L). Baseline electrolytes revealed a sodium of 151 mmol/L (RI 139‐154 mmol/L), a potassium 4.2 mmol/L (RI 3.6‐5.5 mmol/L), and a chloride 111 mmol/L (RI 102‐120 mmol/L). The case was reported to the American Society for the Prevention of Cruelty to Animals, Animal Poison Control Center (ASPCA APCC). Serial serum sodium and glucose monitoring, and measurement of liver enzymes at baseline, 12 and 24 hours from ingestion were recommended. Treatment recommendations included administration of benzodiazepines for seizures; low‐dose acepromazine for agitation; methocarbamol for tremors; and cyproheptadine for the treatment of serotonin syndrome. Dextrose supplementation in the form of an intravenous (IV) continuous rate infusion (CRI), acetylcysteine, and S‐Adenosyl‐L‐methionine (SAMe) were recommended for hepatoprotection. Propranolol was recommended for treatment of tachycardia if the patient was normotensive, and esmolol or atenolol were recommended if the patient became hypertensive and tachycardic. A dose of maropitant citrate was administered (1 mg/kg IV), and the patient was administered 90 mL of liquid activated charcoal (ToxiBan) by mouth. The patient was administered midazolam IV (dose not documented) and initially became less agitated, however subsequently became severely depressed. Thoracic radiographs were performed and no significant abnormalities were identified, with the exception of gastric distension with air and ingesta. Serial BG monitoring revealed the development and persistence of hypoglycemia in the range of 1.665‐4.995 mmol/L (30‐90 mg/dL) (RI 4.4‐6.7 mmol/L, 80‐120 mg/dL). The patient was administered the following boluses of 50% dextrose IV: 1 mL once, 2 mL three times, and 3 mL once, and a 5% dextrose CRI was initiated (rate not recorded). The patient was then referred to the authors’ hospital for continued treatment.

On presentation to the authors’ hospital (day one), the patient was dull to obtunded with minimal response to stimuli and had intermittent generalized tremors. Heart rate was increased at 178 bpm, and the patient was tachypneic, with a respiratory rate of 100 breaths per minute. Thoracic auscultation was within normal limits. Rectal temperature was increased at 39.8°C (103.8°F) (RI 37.5‐39.2°C; 99.5‐102.5°F). Mucous membranes were hyperemic, and the CRT was less than one second. The patient was estimated to be 5% dehydrated. Neurologic examination revealed the following abnormalities: non‐ambulatory tetraparesis with inability to hold the head up, proprioceptive deficits in all four limbs, bilateral mydriasis with absent menace responses and absent pupillary light responses (direct and consensual) (PLR) bilaterally. A Small Animal Modified Glasgow Coma Score (MGCS) of eight was assigned.^4^ Systolic blood pressure (SBP) as measured by Doppler (Doppler Flow Detector, Parks Medical Electronics Inc, Aloha, OR) was 120 mm Hg (range 100‐160 mm Hg). Oxygen saturation as measured by pulse oximetry (Rad‐5, Masimo, Irvine, CA) was 94%‐96% on room air and 98% with flow‐by oxygen administration at 2 L/min. A standard lead II electrocardiogram revealed sinus tachycardia. A venous blood gas (VBG; Siemens Rapid Point 500, Siemens Healthcare Diagnostics, Inc, Tarrytown, NY) was obtained, and mild hyperlactatemia was noted at 2.33 mmol/L (RI <2.0 mmol/L). Blood glucose was 4.385 mmol/L (79 mg/dL) (RI 4.4‐6.7 mmol/L, 80‐120 mg/dL) on an Alpha Trak (Alpha TRAK2, Zoetis Inc, Kalamazoo, MI) glucometer. Baseline electrolytes were within normal limits with the exception of sodium, which was 158 mmol/L (RI 146‐156 mmol/L). A fluorescein stain was performed, and there was mild superficial uptake in both eyes, demonstrating bilateral corneal ulceration. An in‐house prothrombin time (PT) and activated partial thromboplastin time (PTT) were performed; results were 14 and 90 seconds respectively (RI PT 11‐17 seconds, PTT 72‐102 seconds). Thoracic radiographs were performed and no significant abnormalities were identified, with the exception of gastric distension with air and ingesta. The following treatments were prescribed: LRS (Vetivex Veterinary Lactated Ringer's Injection USP, Dechra Veterinary Products, Overland, KS) with 5% dextrose (50% Dextrose Injection USP, Hospira Inc, Lake Forest, IL) supplementation at 40 mL/h (120 mL/kg/d), cyproheptadine (Cyproheptadine, Zydus Pharmaceuticals Inc, Pennington, NJ; 8 mg rectally every 6 hours), methocarbamol (Robaxin Injection USP, West‐Ward Pharmaceuticals, Watontown, NJ; 50 mg/kg IV every 8 hours), acetylcysteine (20% Acetylcystine solution USP, Hospira Inc, Lake Forest, IL; 70 mg/kg IV every 8 hours), Neomycin‐Polymyxin‐Bacitracin ophthalmic ointment (Vetropolycin Bacitracin‐Neomycin‐Polymixin veterinary ophthalmic ointment, Lancaster, SC; ¼ inch strip to both eyes every 8 hours), metoclopramide (Metoclopramide, Hospira Inc, Lake Forest, IL; 2 mg/kg/d IV CRI), and artificial tears (Puralube Veterinary Ointment, Dechra Veterinary Products, Overland Park, KS; applied to both eyes every 6 hours). The patient was monitored for seizure activity, and no seizures were observed. A nasogastric tube (NGT) was placed in order to monitor gastric residual volumes and provide nutritional support if needed.

Approximately 8 hours after admission, the patient developed sustained ventricular tachycardia (VT). A 2 mg/kg bolus of lidocaine (Lidocaine hydrochloride injection 2%, Clipper Distributing Company LLC., St. Joseph, MO) was administered IV, and the rhythm converted to normal sinus rhythm (NSR). Sustained VT was noted again 5 minutes later, and a second lidocaine bolus was administered (2 mg/kg IV), subsequent to which a lidocaine CRI was started (50 mcg/kg/min IV). Over the following 12 hours, the lidocaine CRI was tapered to 45 mcg/kg/min IV and then 40 mcg/kg/min IV with no additional VT. The patient was euglycemic to hyperglycemic (range 5.385‐9.879 mmol/L, 97‐178 mg/dL on glucometer), and the dextrose CRI was decreased to 2.5%. A chemistry panel performed at 12 hours post‐ingestion revealed an increase in ALT of 248 IU/L (RI 18‐121 IU/L); AST measurement was unavailable on this in‐house panel. The remainder of the values were within reference intervals. Repeat VBG demonstrated resolution of the hyperlactatemia.

On day 2, the patient was responsive to sound and physical stimuli, and ambulatory with a normal gait. Anisocoria was noted (the right pupil was miotic, the left pupil was normal). Menace responses and PLRs were present bilaterally. Hyperthermia, tachypnea, and tachycardia had resolved. No additional arrhythmias were documented, and the lidocaine CRI was weaned and discontinued. Oxygen saturation was consistently 98%‐100% on room air, so supplemental oxygen was discontinued. No additional muscle tremors were noted, and methocarbamol and cyproheptadine were discontinued. Acetylcysteine was continued (70 mg/kg IV every 8 hours). The patient began eating consistently. A chemistry panel revealed the following abnormalities: ALT 268 IU/L (RI 18‐121 IU/L), AST 1346 IU/L (RI 16‐55 IU/L), total protein (TP) 51 g/L (5.1 g/dL) (RI 55‐75 g/L, 5.5‐7.5 g/dL), albumin 25 g/L (2.5 g/dL) (RI 27‐39 g/L, 2.7‐3.9 g/dL), and creatine kinase (CK) 27 795 IU/L (RI 10‐200 IU/L). The remainder of chemistry values were within normal limits. Electrolytes were within normal limits, with the exception of sodium which was below reference range‐ 138.2‐144.0 mmol/L (RI 146‐156 mmol/L).

On day 3, 48 hours after ingestion of Natrol 5‐HTP, the patient was bright and alert. Vital signs were within normal limits, including blood pressure and oxygen saturation. The patient was ambulatory without ataxia, and the anisocoria had resolved. Menace response and PLRs were present bilaterally. The patient was intermittently trembling but only when handled—believed to be related to anxiety. Dextrose supplementation was discontinued, and the patient remained euglycemic. Denamarin (Denamarin, Nutramax Laboratories, Veterinary Sciences Inc, Lancaster, SC; 225 mg by mouth every 24 hours) was added, and the patient was transitioned to oral metoclopramide (Metoclopramide 5 mg tablets, Teva Pharmaceuticals USA, Inc, North Wales, PA; 2.5 mg every 6 hours). Repeat biochemistry values at that time revealed the following: ALT 321 IU/L (RI 18‐121 IU/L), AST 326 IU/L (RI 16‐55 IU/L), TP 53 g/L (5.3 g/dL) (RI 55‐75 g/L, 5.5‐7.5 g/dL), albumin 26 g/L (2.6 g/dL) (RI 27‐39 g/L, 2.7‐3.9 g/dL), and CK 2369 IU/L (RI 10‐200 IU/L). The remainder of values were within normal limits.

The patient was discharged for continued monitoring at home with the following medications: Denamarin 225 mg by mouth every 24 hours, metoclopramide 2.5 mg by mouth every 8 hours, and Neomycin‐Polymyxin‐Bacitracin ophthalmic ointment ¼ inch strip to both eyes every 8 hours. Five days following discharge, neurologic examination was normal and chemistry values were within reference intervals. Denamarin was continued, and recheck bloodwork was recommended one month later.

## 3 DISCUSSION

Five‐hydroxytryptophan and xylitol are found separately in numerous readily accessible products, and both have been documented to cause serious adverse effects or fatality in dogs following accidental ingestion.^1^, ^3^, ^5^ This is the first report of simultaneous signs of toxicosis in a dog ingesting one product containing both compounds.

Five‐hydroxytryptophan has been reported to be fatal at a dose of 128 mg/kg according to a retrospective evaluation of cases in the ASPCA APCC database.^1^, ^6^ A minimum lethal dose has not been established, however, in a review of 21 cases published by Gwaltney‐Brant et al,^1^ the lowest dose at which clinical signs were reported was 23.6 mg/kg. Clinical signs of toxicity manifest as serotonin‐like syndrome, and include neurologic signs such as seizures, depression, tremors, hyperesthesia, ataxia, paresis, disorientation, mydriasis, and coma; and gastrointestinal signs such as vomiting, diarrhea, abdominal pain, hypersalivation, flatulence, and bloat. Other symptoms such as hyperthermia, weakness, vocalization, arrhythmias, cyanosis, recumbency, dyspnea, and hypothermia have been reported.^1^, ^2^ The patient in the current case report exhibited many of the signs listed above, consistent with serotonin‐like syndrome.

Xylitol has a wide margin of safety in most species. In people, doses greater than 130 g/d of xylitol can cause diarrhea, but no other consequences have been reported.^3^ Jerzsele et al^7^ evaluated the effects of xylitol on cats and found no significant changes to hematologic and biochemical parameters when cats were administered 0.1, 0.5, and 1.0 g/kg. In dogs, ingestion of xylitol causes a dose‐dependent increase in plasma insulin concentration which subsequently causes hypoglycemia.^3^, ^5^ Insulin release is roughly 2.7‐7 times greater with ingestion of xylitol when compared to ingestion of the equivalent amount of glucose.^8^ According to the ASPCA APCC, hypoglycemia has been reported in dogs with ingestions of less than 0.1 g/kg.^5^ Another unique effect of xylitol in dogs is the development of hepatotoxicity, and increases in ALT and AST have been identified in dogs exposed to xylitol concentrations of greater than 1.0 g/kg.^9^ Increases in total bilirubin and GGT, and prolongations in PT and PTT have also been reported. Acute hepatic failure was described in a single case report^8^ and a case series.^10^ In the former report, the patients’ liver enzymes (LES) normalized and a full recovery occurred following supportive care. In the latter report, five dogs died after developing progressive coagulopathies and clinical deterioration.^10^ The lowest estimated xylitol dose associated with hepatic failure in dogs, as reported by the ASPCA APCC, is 0.5 g/kg.^5^ Two possible mechanisms for xylitol‐induced hepatic necrosis have been proposed.^8^, ^10^ The first is that depletion of adenosine triphosphate (ATP) may result in the inability of liver cells to perform normal cellular functions including protein synthesis and maintenance of membrane integrity, which ultimately leads to cellular necrosis. The second is that metabolism of xylitol results in high concentrations of cellular nicotinamide adenine dinucleotide, which leads to production of reactive oxygen species that damage cellular membranes and cause necrosis.^10^ Conversely, DuHadway et al^5^ reported 192 dogs that ingested xylitol, very few of which developed increases in LES, and none of which developed hepatic failure, suggesting that hepatotoxicity in dogs following xylitol ingestion may be idiosyncratic.

A number of biochemical abnormalities were observed in the case reported here. In addition to hypoglycemia, increases in LES (ALT, AST) and CK were noted, and a decrease in total protein and albumin was seen. Alanine aminotransferase (ALT) is a cytosolic enzyme that is released by hepatic cells secondary to hepatic injury or necrosis.^8^, ^11^ Generally, ALT levels increase within 12 hours of a toxic insult, and peak within 24‐48 hours if ongoing injury does not occur.^8^, ^11^ Muscle necrosis has also been identified as a source for increased serum ALT activity, and levels can increase secondary to muscle tremors seen in serotonin syndrome.^11^ Aspartate aminotransferase (AST) is primarily released from hepatic, muscle, and red blood cells, although it is found in most tissues.^11^ The serum half‐life is shorter than that of ALT, and clearance generally occurs within 12 hours in the absence of ongoing injury.^11^ Generally, AST is evaluated in conjunction with CK, which is released by injured muscle cells, when assessing whether elevation is primarily caused by hepatic disease or muscle damage.^11^ Increased circulating serum CK levels are caused by degenerative or necrotizing muscle injury, and CK is the most sensitive serum enzyme indicator of striated muscle damage. In people and dogs with serotonin syndrome, tremors, hyperreflexia, spontaneous clonus, and muscle rigidity could all contribute to release of CK.^1^, ^12^ As such, the increase in ALT, AST, and CK observed in the patient reported here could have been secondary to a combination of hepatocellular injury induced by xylitol, and muscle activity secondary to serotonin‐like syndrome. During hospitalization, hypoproteinemia characterized by hypoalbuminemia was also observed in this patient. Albumin is a negative acute phase protein and can decrease with acute tissue injury or inflammation. It also is produced by the liver and can be decreased with hepatic insufficiency.^11^ Moreover, a relative decrease of measured albumin may be seen in patients receiving intravenous fluid therapy due to dilution, which may contribute to a lower measured albumin in hospitalized patients. The cause of the hypoalbuminemia in this patient was likely due to a combination of the above, and it resolved during the course of hospitalization.

Treatment of veterinary patients with serotonin‐like syndrome clinical signs generally includes administration of IVF, anti‐emetics, treatment of hypertension and arrhythmias, and treatment of neurologic signs such as seizures and tremors. Cyproheptadine, a 5HT_2A_ antagonist, has been reported to have variable success for treatment of serotonin syndrome in humans.^12^ Cyproheptadine binds serotonin receptors to competitively prevent serotonin molecules from binding to receptors. Treatment with cyproheptadine is generally administered only while the patient has clinical signs of serotonin syndrome, as was the case with the patient reported here. Cyproheptadine is not available in a parenteral formulation, however, it can be administered to companion animals by crushing and administering via NGT, or by administration per rectum.^12^ Treatment of xylitol toxicosis includes administration of IVF containing 2.5%‐5% dextrose. Glucose is required for ATP synthesis, thus administering dextrose may help preserve ATP levels thus minimizing hepatic necrosis. It has been suggested that administration of dextrose can be hepatoprotective in dogs ingesting more than 0.5 g/kg of xylitol.^3^ Hepatoprotectants such as SAMe, Denosyl and Marin, milk thistle, and acetylcysteine have been recommended, although efficacy against development of hepatic necrosis secondary to xylitol toxicosis has not yet been established.^3^

Treatment considerations for toxicities in general include decontamination via emesis and gastric lavage, and administration of adsorbents such as activated charcoal. Additional treatments such as intravenous lipid emulsion therapy (ILE), and extracorporeal techniques such as hemodialysis (HD) and hemoperfusion have also been investigated for treatment of certain toxic compounds. Activated charcoal is an adsorbent that binds chemicals and drugs via hydrogen binding, and prevents or decreases absorption.^13^ In the human literature, activated charcoal has been described for use in treatment of serotonin‐like syndrome,^14^ however to the authors’ knowledge, there are no reports of the use of activated charcoal in veterinary patients exhibiting serotonin‐like syndrome. In vitro studies have shown low binding of xylitol to activated charcoal, likely due to the rapid absorption of xylitol after ingestion (30‐60 minutes).^14^ A toxicology study performed by Cope demonstrated that the mean percentage of activated charcoal binding to xylitol following in vitro incubation was low, ranging from 8% to 23%.^14^ As such, there is no evidence for the use of activated charcoal in veterinary patients suffering from xylitol toxicosis. Moreover, due to the need for oral administration, the use of activated charcoal in patients that are neurologically inappropriate (as can be the case in both xylitol toxicity and serotonin‐like syndrome) is contraindicated because aspiration can occur. ILE has been shown to be an effective antidote for intoxication caused by numerous lipophilic agents. The exact mechanism of action remains unknown, however, it is suspected that ILE serves as a “lipid sink” for fat‐soluble compounds, reducing the amount of free drug in circulation, thereby reducing the drug's toxic effects.^13^, ^15^ Other proposed mechanisms include the “change in energy theory,” in which ILE provides enough fatty acid to facilitate myocardial fatty acid utilization, and/or inhibit endothelial nitric oxide synthase which decreases nitric oxide‐induced vasodilation.^15^ Intravenous lipid emulsion therapy has been described for the treatment of citalopram and venlafaxine overdoses in human literature, as both selective serotonin reuptake inhibitor (SSRI) and selective norepinephrine reuptake inhibitor (SNRI) drugs are lipophilic,^16^, ^17^ and it could be considered for veterinary cases of serotonin‐like syndrome secondary to toxicity. Xylitol is not lipophilic, therefore ILE alone would not be effective for the treatment of xylitol toxicity. A study by Roboz et al^18^ in 1990 evaluated serum levels of xylitol and other polyols before, during, and after HD, and found that no significant serum level changes were observed in these human patients, inferring these modalities would be of limited use for xylitol toxicity. Conversely, given the low molecular weight and low protein binding of 5‐HTP, the use of these therapies may be of benefit in serotonin toxicities. Kamo et al^19^ evaluated the efficacy and pharmacokinetics of fluvoxamine maleate, an SSRI, in patients undergoing HD, and found a 22% reduction in serum drug levels. To the authors knowledge, there are no reports to date of the use of HD to treat serotonin‐like syndrome in human or veterinary patients.

In conclusion, both xylitol and 5‐HTP have been described to cause toxicity, even fatality, in dogs, and increased awareness of over‐the‐counter products containing both these compounds is needed. Fortunately, in spite of the high doses ingested, with prompt treatment and supportive care, the patient in this report survived to discharge.

## CONFLICT OF INTEREST

None declared.

## AUTHOR CONTRIBUTION

JO: contributed as primary manuscript author. TB: heavily involved in manuscript editing. MG: managed patient and involved in editing manuscript.
